# Production of antibacterial compounds by a Steely hybrid polyketide synthase in *Dictyostelium*

**DOI:** 10.1002/2211-5463.70124

**Published:** 2025-10-08

**Authors:** Tomoaki R. Yamashita, Toyonobu Usuki, Robert R. Kay, Tamao Saito

**Affiliations:** ^1^ Graduate School of Science and Technology Sophia University Tokyo Japan; ^2^ Faculty of Science and Technology Sophia University Tokyo Japan; ^3^ MRC Laboratory of Molecular Biology Cambridge UK

**Keywords:** antibacterial activity, chlorinated dibenzofuran, *Dictyostelium*, polyketide, Steely

## Abstract

Ecological interactions in the soil are often mediated by small molecules, which can later become valuable drugs. The cellular slime mould *Dictyostelium discoideum* is a soil microbe with a life cycle consisting of unicellular (amoeba) and multicellular phases (fruiting bodies). After *Dictyostelium* amoebae have consumed all available bacteria, they form stalked fruiting bodies to aid dispersal of the spores. The dying stalk cells repurpose a hybrid polyketide synthase to make abundant chlorinated metabolites, which persist in their fruiting bodies. The most abundant of the chlorinated metabolites, CDF‐1, is a chlorinated dibenzofuran, which was shown to be an effective antimicrobial, being roughly as potent as ampicillin. Here, we identify CDF‐2 and ‐3 by purification, followed by MS and NMR, after increasing their yields by using producer species and growth condition optimisation. Similar to CDF‐1, CDF‐2 and ‐3 are chlorinated dibenzofurans and exhibit more potent antibacterial activity against Gram‐positive bacteria than ampicillin. We propose that the ecological function of CDF‐2 and ‐3 is to protect the dormant spores from degradative bacteria.

## Graphical abstract

By selecting appropriate production species under optimised culture conditions, new chlorinated dibenzofuran compounds, which previously could not be analysed in sufficient quantities, were identified from cellular slime mould fruiting bodies. The strong antibacterial activity exhibited by these compounds suggests a link to the ecological function of protecting spores in fruiting bodies.

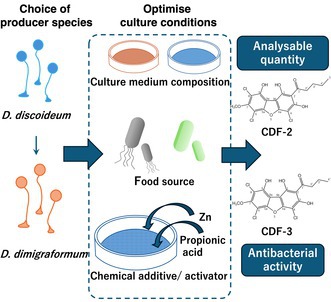

**Abbreviations**
MIC: minimum inhibitory concentration
PKS: polyketide synthase

Soil is a complex environment with much of its biomass made up of bacteria, fungi, protozoa and nematodes. Soil microorganisms synthesise a great variety of natural products to communicate with each other and as weapons of defence and offence [1, 2, 3]. This leads to complex survival strategies that are important for the health and productiveness of the soil. These natural products, also known as secondary metabolites, have diverse chemistries but can be very potent and specific effectors of particular molecular processes and so have become valuable pharmaceutical resources for antibiotics, anticancer drugs and immunosuppressants, as well as pesticides.

The cellular slime mould *Dictyostelium discoideum* is a soil microbe with a life cycle consisting of unicellular and multicellular phases. In the unicellular stage, the cells live as amoebae and eat bacteria but when these are depleted, they aggregate into multicellular organisms, taking approximately 24 h to form stalked fruiting bodies consisting of stalk cells and spores. Spore cells are resistant and remain viable for long periods, hatching when they encounter suitable growth conditions. Stalk cells die, with their altruism considered to aid the dispersal and survival of the spores [4].

Genomic analysis of *Dictyostelium* has revealed a great potential for producing secondary metabolites. The *D. discoideum* genome contains 45 polyketide synthase (*PKS*) genes and nine terpene synthase genes [5, 6, 7]. These considerations, including the complex ecology of the habitat and the presence of many genes for synthesising secondary metabolites, suggest that *Dictyostelieum* may be an untapped resource for novel bioactive compounds [7, 8].

The first polyketide to be identified in *Dictyostelium* was DIF, a chlorinated alkyl phenonone, which is used for communication between the amoebae during their developmental phase [9]. The skeleton of DIF is made by a novel type of PKS consisting of a fusion of a type I and type III (chalcone synthase) PKS enzymes [10]. This ‘Steely’ fusion is found across Dictyostelid species but not elsewhere. The backbone of DIF made by SteelyB (StlB) is then decorated by chlorination (ChlA) and methylation (DmtA) [10, 11, 12].

Early labelling experiments with ^36^Cl‐revealed an unexpected complexity: apart from DIF and its metabolites, which peak during the middle of development, they also detected another much more abundant group of chlorinated compounds made only at the end of development and for several days afterwards [13]. Again, unexpectedly, these compounds are also made by the same PKS (StlB) that makes DIF, as well as the same chlorinating enzyme. To achieve this product‐switch the cells express StlB in stalk cells instead of prespore cells and it is cleaved to release the chalcone synthase domain, which is responsible for synthesing the new compounds [14]. Consistent with the conservation of StlB, abundant chlorinated compounds are made in late development across Dictyostelid species [15, 16, 17]. This speaks for them having a conserved ecological function, perhaps aiding spore dispersal or protecting the spores in some way.

The most abundant of the late chlorinated compounds, CDF‐1, was recently identified as a chlorinated dibenzofuran [14]. It was also found to be an effective antimicrobial, being roughly as potent as ampicillin, suggesting that its function is to protect the spores from bacterial attack. The lower abundance of the remaining chlorinated compounds precluded their immediate identification, although MS suggested they are related to CDF‐1.

Hypothesising that these CDF compounds might be functionally specialised and thus their production elicited more strongly in different species or under particular environmental conditions, we explored these parameters to increase their production. In this we succeeded and were able to identify CDF‐2 and ‐3 and show that they are closely related to CDF‐1 and likewise potent antibacterials.

## Materials and methods

### *Dictyostelium* species and culture conditions

In the present study, we used five species of *Dictyostelium*. Four of these, *Dictyostelium dimigrformum, Dictyostelium citrinum, Dictyostelium intermedium* and *Dictyostelium firmibasis*, were purchased from the NBRP (https://nenkin.nbrp.jp). *D. discoideum* strain V12M2 cells were used to compare culture conditions.

For structural determination, all *Dictyostelium* species, including *D. discoideum*, were cultured on a 5LP plate with *Escherichia coli* B/r as a food source. Culture on 5LP medium (lactose 5 g, proteose peptone 5 g, agar 15 g per 1 L of 5.4 mm Na_2_HPO and 21.1 mm KH_2_PO_4_) was performed using large Petri dishes measuring 25 cm square. After the cells had been cultivated on the culture medium for 8 days and had completely formed fruiting bodies, they were scraped off using a glass slide. Table 1 shows the wet weight of the fruiting bodies and the yield of the CDFs from each species. Because *D. dimigraformum* gave the highest yield, its culture conditions were further modified to maximise the yield. To activate PKS, 0.5 mm ZnCl_2_ was added. Additionally, 0.1 g·L^–1^ of sodium propionate was added to increase the yield of CDF with odd‐numbered alkyl chains.

**Table 1:** CDF yield of different *Dictyostelium* species belonging clade 4C. The wet weight of fruiting bodies from 20 Petri dishes of 5LP agar used to cultivate each cellular slime mould species from clade 4C and the yield of each CDF. *D. dimigraformum* produced the highest yield.

Species	*D. dimigraformum*	*D. citrinum*	*D. firmibasis*	*D. discoideum*	*D. intermedium*
Wet weight (g)	72.32	49.73	50.48	38.81	62.62
Ethanol extraction (g)	1.708	1.655	1.581	1.199	2.024
Two phase separation (mg)	176.8	128.8	102.7	179.4	124.2
Silica gel c.c. (mg)	14.8	28.9	42.5	54.7	22.1
CDF‐1 (mg)	3.4	1.7	0.9	2.5	2.5
CDF‐2 (mg)	1.0	0.6	0.3	0.8	1.0
CDF‐3 (mg)	2.6	2.2	0.5	0.1	0.1
Total CDFs (mg)	7.0	4.5	1.7	3.4	3.6

### Purification and analysis of CDFs

Purification of CDFs was performed by slightly modifying a previously reported method [14]. The CDFs were monitored using reverse‐phase HPLC, which was performed at 1 mL·min^−1^ with a gradient of 50–80% acetonitrile containing 1% acetic acid for 50 min. Detailed structural analyses of purified CDF‐2 and ‐3 were performed using MS (AccuTOF LC‐Express; JEOL, Tokyo, Japan) and NMR (JNM‐ECA 500; JEOL).

### Antibacterial activity

*E. coli B/r*, *Bacillus subtilis* and *Klebsiella aerogenes*, which are used as food for cellular slime moulds, were used for the antibacterial activity tests.

*E. coli* (NBRC14249), *Pseudomonas fluorescens* (ATCC 13525), *B. subtilis* (ATCC 6051) and *Staphylococcus epidermidis* (ATCC 14990) were purchased from NBRC (https://www.nite.go.jp/nbrc/cultures/nbrc) and used for antibacterial activity tests.

The antibacterial activity was measured using the Mueller–Hinton liquid dilution method. Two‐fold serial dilutions of ampicillin (positive control) and CDF‐1 were added to 96‐well plates. The highest concentration of ampicillin sodium was 128 μg·mL^−1^ (ampicillin concentration 120 μg·mL^−1^). The highest concentration of CDF‐1, ‐2 and ‐3 was 100 μg·mL^−1^. Two‐fold serial dilutions of 2% dimethylsulfoxide in saline were used as negative controls.

The diluted samples with added bacteria were cultured in a 96‐well plate at 37 °C for 16 h, except for *P. fluorescens*, which was cultured at 30 °C. The bacterial growth in each well was confirmed using microscopy. The highest dilution at which growth inhibition was observed was defined as the minimum inhibitory concentration (MIC) based on three to four independent experiments.

## Results

### The presence of two CDF‐1‐related compounds in the *D. discoideum* fruiting body

**Fig. 1:**
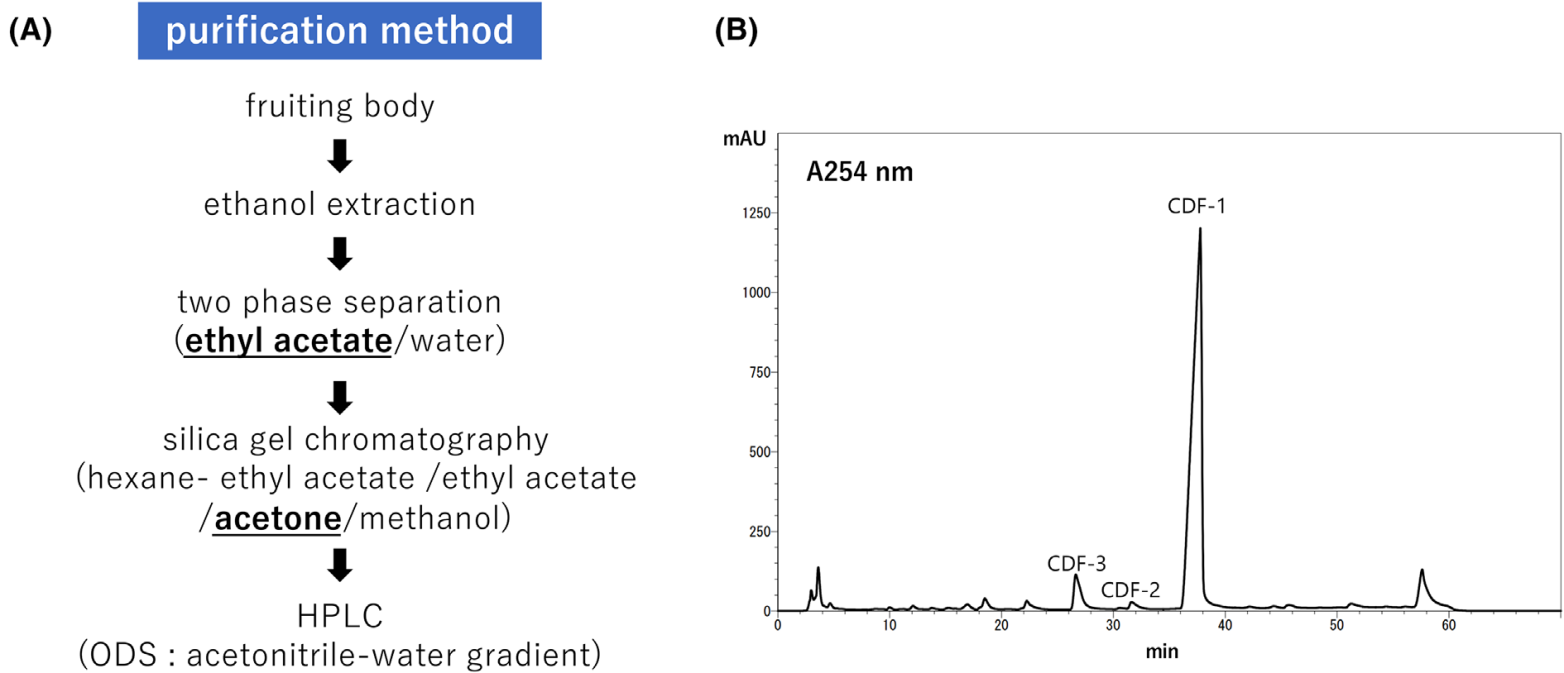
Purification method and HPLC profile of CDFs from *D. discoideum*. Flowchart of the CDF purification strategy (A). *D. discoideum* fruiting bodies cultured on SM agar containing *K. aerogenes* were collected and purified according to the flowchart. The acetone fraction obtained using silica column chromatography was subjected to a 50–80% acetonitrile gradient using reverse‐phase HPLC (B).

The hybrid‐type PKS SteelyB, together with the chlorinating enzyme ChlA, synthesise several chlorinated compounds in the stalk cells of *Dictyostelium* fruiting bodies. Our previous purification scheme allowed us to resolve three of these but only CDF‐1 was obtained in sufficient quantities for identification [14]. Figure 1A shows the purification procedure for CDF‐1 and Fig. 1B shows the HPLC profile of the acetone fraction of the silica column. Among the three compounds shown in Fig. 1B, CDF‐1 has already been identified. Based on mass spectral analysis, both compounds CDF‐2 and ‐3 contained three chlorines, and the estimated compositional formula suggests that they are related to CDF‐1.

To obtain analysable quantities of compounds CDF‐2 and ‐3, we attempted to increase their production using the HPLC profile of the extracts to monitor success as we varied conditions. First, we tested different culture conditions, as is also recommended in one of the ‘one strain of many compounds’ (i.e. OSMAC) strategies [18]. Second, we tested whether each compound is synthesised in greater amounts by other *Dictyostelium* species closely related to *D. discoideum* [19]. Finally, we tested whether production could be increased by adding biosynthetic precursors or low concentrations of heavy metals, such as zinc or cadmium, which may activate polyketide synthesis [20, 21, 22, 23, 24, 25].

### A two‐member culture with *E. coli* is suitable for producing CDF‐2 and ‐3

**Fig. 2:**
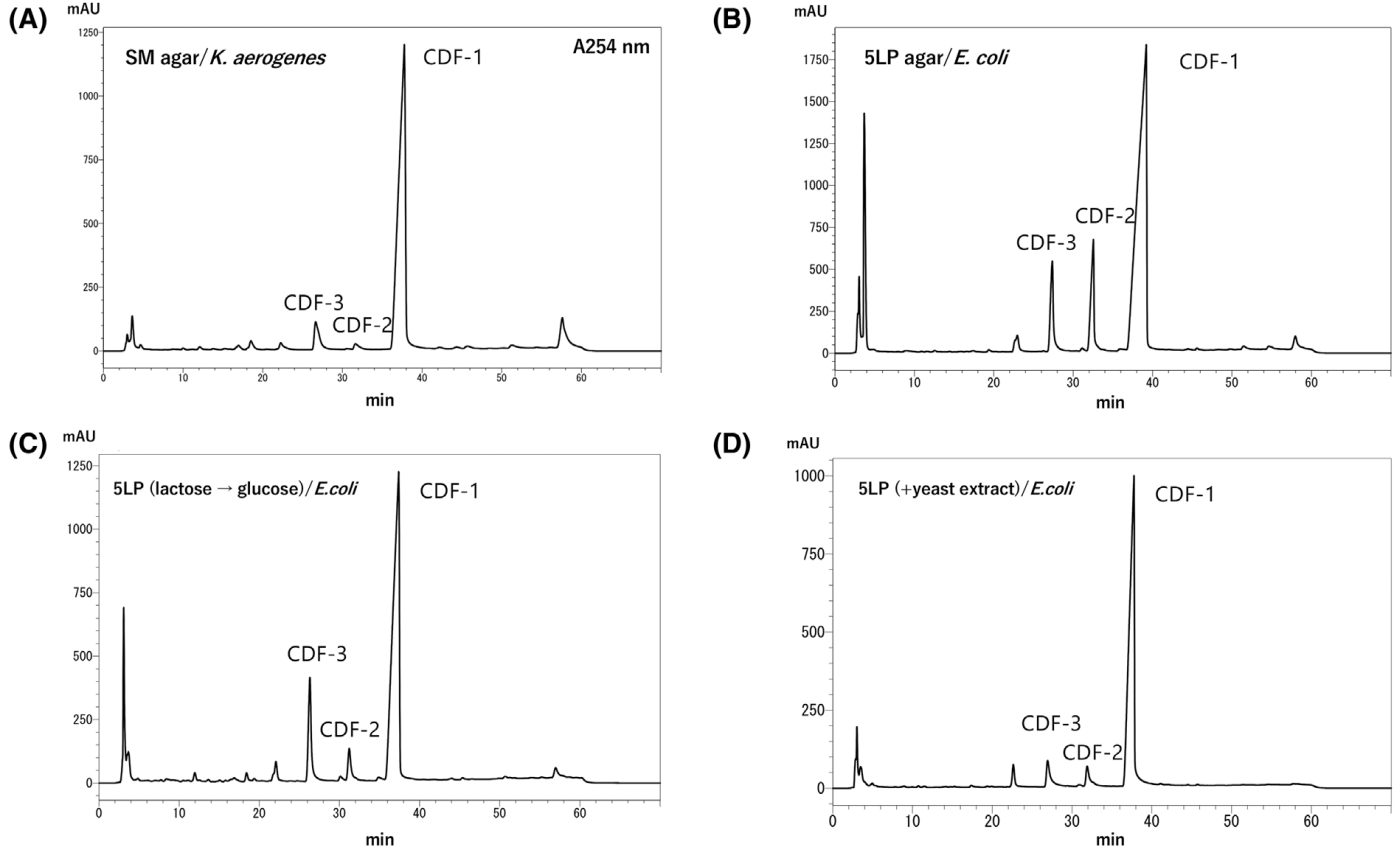
HPLC profile of CDFs produced by *D. discoideum* cultured under different conditions. *D. discoideum* cultured on SM agar with *K. aerogenes* as food (A). *D. discoideum* cultured on 5LP agar with *E. coli* (B). *D. discoideum* cultured on 5LP agar medium in which the lactose in the medium has been replaced with glucose (C). *D. discoideum* cultured on 5LP agar medium containing yeast extract (D). The HPLC analysis of the CDFs obtained under each condition was repeated three times. Representative HPLC profiles for each condition are shown.

*Dictyostelium* cells can be grown in bulk in axenic culture, but because the CDFs are extracted from mature fruiting bodies, the cells would then have to be plated on agar for development. A simpler procedure, which we adopted, is to grow *Dictyostelium* cells in association with bacteria on nutrient agar plates, where they will also develop once the bacteria are exhausted. Figure 2 shows the HPLC profiles obtained under different incubation conditions. The commonly used SM agar is a rich medium containing peptone, yeast extract and glucose, producing a thick lawn of *K. aerogenes*. Under these conditions, CDF‐1 is abundant; however, CDF‐2 and ‐3 were present in low quantities, as indicated by small peaks in the HPLC profile (Fig. 2A). Therefore, LP agar, which contains lactose and peptone and is usually used for two‐member cultures with *E. coli* B/r where it forms relatively thin lawns, was used to test relatively poor nutritional conditions. The HPLC profile showed that now the peaks of CDF‐2 and ‐3 were larger than the other compounds (Fig. 2B).

Next, using LP agar as the basis, some of the ingredients were changed. As shown in Fig. 2C, lactose was substituted to glucose and, as shown in Fig. 2D, yeast extract was added. The results showed that both conditions resulted in smaller peaks for CDF‐2 and ‐3. Glucose and yeast extracts did not promote the synthesis of CDF‐2 and ‐3.

### *Dictyostelium* species in the same clade as *D. discodeum* synthesise CDFs in different proportions

Over 100 species of cellular slime mould have been identified, with molecular phylogenetic analysis using small subunit ribosomal DNA dividing them into four major groups [26]. Group 4 to which *D. discoideum* belongs is assumed to be the most advanced.

**Fig. 3:**
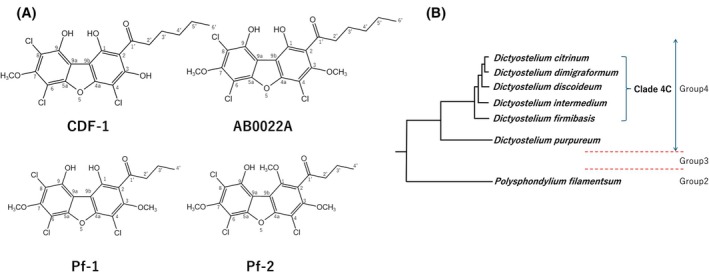
Structure of identified chlorinated dibenzofuran compounds and phylogenetic relationships of cellular slime moulds synthesising them. (A) CDF‐1 was identified in *D. discoideum* [14]. AB0022A was identified from *D. purpreum* and Pf‐1 and 2 were identified from *Polysphondylium filamentosum* [27, 28]. (B) A diagram showing the phylogenetic relationships of cellular slime moulds synthesising the compounds indicated in (A). The original phylogenetic tree is by Schild *et al*. [19]. *P. filamentosum* belongs to group 2, whereas all others belong to group 4. Within group 4, all except *D. purpureum* belong to clade 4C and are considered to be closely related species.

Three chlorinated dibenzofurans, apart from CDF‐1, have previously been reported from cellular slime moulds (Fig. 3A). *D. purpureum*, which belongs to group 4, produces AB0022A, a chlorinated dibenzofuran with the same structure as CDF‐1, except for a methyl group in a different position [27]. *Polysphondylium filamentosum* belonging to group 2, synthesises Pf‐1 and Pf‐2, which have different alkyl side chain lengths and methyl group positions compared to CDF‐1 [28]. A phylogenetic tree of cellular slime moulds synthesising dibenzofuran compounds is shown in Fig. 3B.

**Fig. 4:**
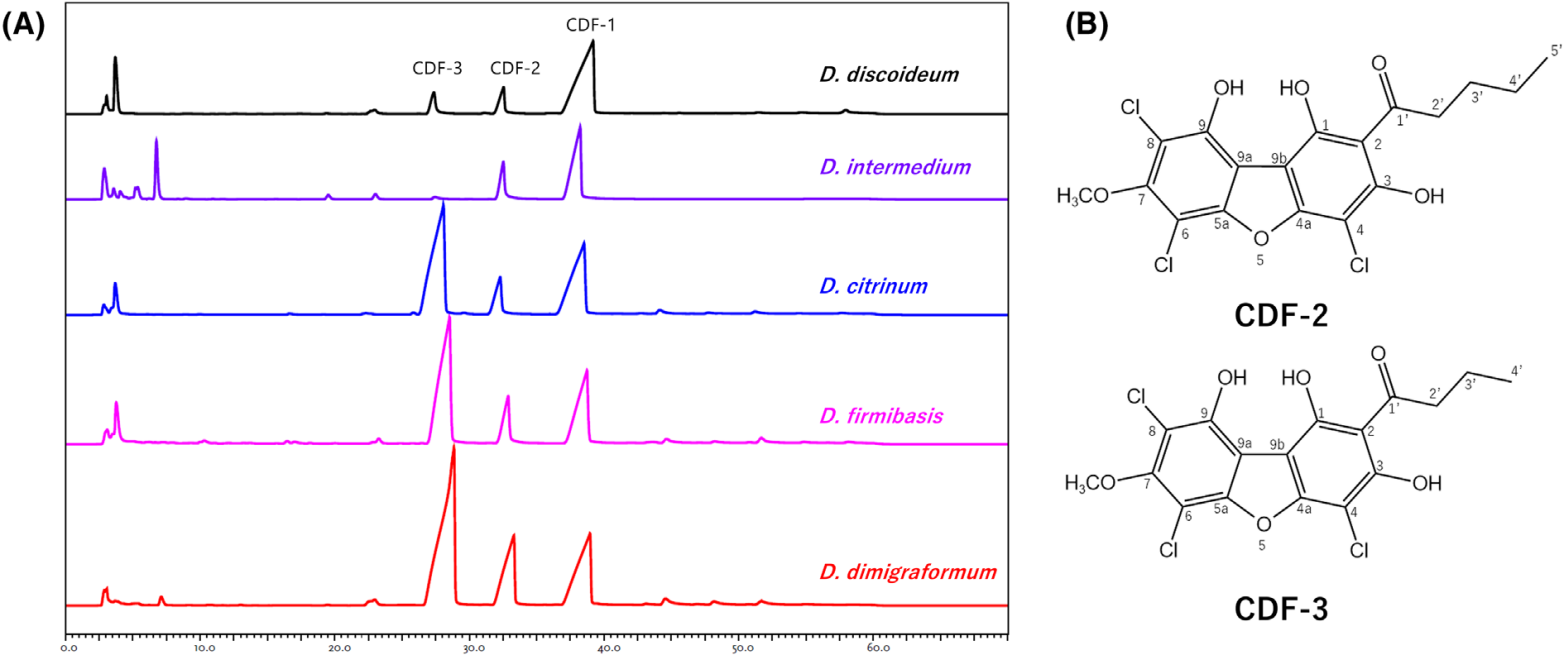
Species closely related to *D. discoideum* also make CDF‐2 and 3. HPLC analysis of the acetone fractions obtained from *Dictyostelium* species closely related to *D. discoideum* (group 4C), which were cultured under the same conditions on 5PL agar with *E. coli*. (A). Structures of chlorinated dibenzofuran compounds CDF‐2 and ‐3 (B). The HPLC was performed three times, and a representative HPLC profile is shown.

Genome analysis revealed that polyketide synthases have diversified considerably between slime mould species except for the two Steely enzymes, which are much more conserved [15, 16, 17]. We therefore determined to what extent the product of the SteelyB enzyme, chlorinated dibenzofuran, is also conserved across species. According to the molecular phylogenetic tree, shown in Fig. 3B. *D. discoideum* belongs to clade 4C of group 4 [19]. Therefore, we examined whether all five species in this clade produced CDFs. Figure 4A shows the HPLC profile of the acetone fraction from the silica column for each *Dictyostelium* species. We found that all five species in clade 4C produced three CDFs, but at different ratios. All CDF samples were verified not only by HPLC retention time, but also by mass spectrum, and confirmed to be CDF‐1, ‐2 and ‐3, respectively (Fig. S1). When comparing the HPLC profiles, *D. firmibasis*, *D. dimigraformum*, and *D. citrinum* had relatively large peaks of CDF‐3 and small peaks of CDF‐1. However, *D. intermedium* was similar to *D. discoideum*, with a large CDF‐1 peak and very small peaks of CDF‐2 and ‐3. We conclude that CDF‐1, ‐2 and ‐3 are all made by this group of Dictyostelids but in differing proportions. Table 1 shows the yield at each purification stage, from the wet weight to the final purified product. Based on these results, we used *D. dimigraformum* to determine the structures of CDF‐2 and ‐3.

### Enhancing CDF production in *D. dimigraformum*

**Fig. 5:**
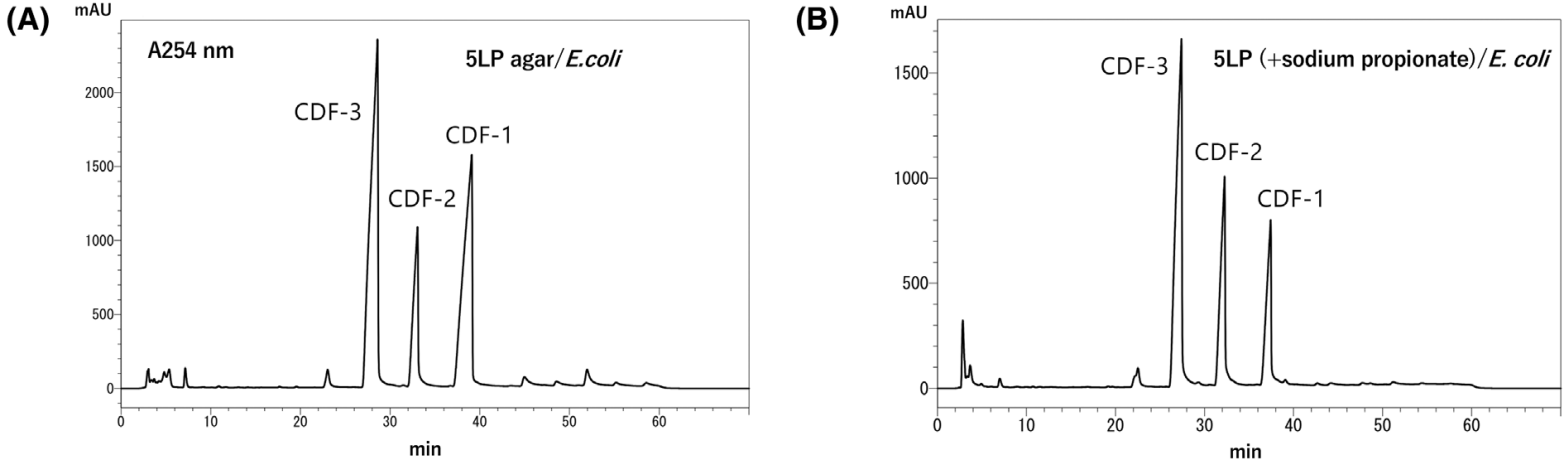
Propionate stimulates CDF‐2 production by *D. dimigraformum*. HPLC profiles of acetone fraction from *D. dimigraformum* grown in with *E. coli* on 5LP agar (A) Or with sodium propionate (B). HPLC was performed three times under each set of conditions, with representative HPLC profiles shown for (A) and (B), respectively.

We next asked whether the yields of CDF‐2 and ‐3 from *D. dimigraformum* could be increased by further modifying the culture conditions. Cells of *D. dimigraformum* were cultured with *E. coli* on LP agar medium as a two‐member culture. Figure 5A shows the HPLC profile. Since CDF‐2 is predicted to have an odd number of carbons in its alkyl side chain, propionic acid was added to the LP medium in an attempt to increase its yield [29]. This met with some success (Fig. 5B). The addition of heavy metals, such as zinc or cadmium, can activate polyketide synthesis [20, 21, 22, 23, 24, 25]; therefore, we added zinc to the LP culture medium and found that the yield increased even further. Thus, by adding propionic acid and zinc to the LP medium and culturing *D. dimigraformum* with *E. coli*, we obtained CDF‐2 and ‐3 in sufficient quantities for NMR analysis (Table 2).

**Table 2:** The yield of each CDF from *D. dimigraformum* with different culture conditions. *D. dimigraformum* was cultivated in 5LP without any additives, with only zinc chloride added, with only sodium propionate added, and with both additives. The wet weight of fruiting bodies collected and the yield of CDFs, as well as the proportion of CDF‐2 in the total amount of CDF‐1, ‐2 and ‐3, are summarised.

Culture condition	5LP	5LP + ZnCl_2_	5LP + sodium propionate	5LP + ZnCl_2_ + sodium propionate
Culture plate	20	10	10	86
Wet weight (g)	72.32	32.70	35.69	295.14
Ethanol extraction (g)	1.708	0.705	0.673	7.436
Two phase separation (mg)	176.8	100.1	139.4	1117.0
Silica gel c.c. (mg)	14.8	11.2	14.2	121.0
CDF‐1 (mg)	3.4	2.0	0.8	12.9
CDF‐2 (mg)	1.0	0.8	0.7	8.9
CDF‐3 (mg)	2.6	1.4	0.8	18.7
Total CDFs/10 plate (mg)	3.5	4.2	2.3	4.7
Ratio of CDF‐2	14%	19%	30%	22%

### Structure elucidation of CDF‐2 and ‐3

MS of CDF‐2 (electrospray ionisation‐MS using a JEOL JMS‐700) in negative ion mode showed four molecular ion peaks at *m*/*z* 431, 433, 435 and 437 at a ratio of 27 : 27 : 9 : 1, suggesting that CDF‐2 contains three chlorine atoms. High‐resolution electrospray ionisation‐MS measurements in negative ion mode provided *m*/*z* 430.98560, consistent with the molecular formula for CDF‐2 of C_18_H_14_Cl_3_O_6_.

MS of CDF‐3 in the negative ion mode also showed four molecular ion peaks at *m*/*z* 417, 419, 421 and 423 in a ratio of 27 : 27 : 9 : 1, and high‐resolution electrospray ionisation MS measurements in the negative ion mode provided *m*/*z* 416.96995, which is consistent with the molecular formula for CDF‐3 of C_17_H_12_Cl_3_O_6_. The full chemical structure was elucidated using proton NMR (^1^H‐NMR) and carbon‐13 NMR (^13^C‐NMR) (JEOL JNM‐ECA 500) and compared with the known CDF‐1 and is summarised as follows (Table 3).

**Table 3:** Summary of 1H and 13C NMR spectra in dimethylsulfoxide‐d_6_ of CDF‐1, ‐2 and ‐3.

	CDF‐1		CDF‐2		CDF‐3	
	^1^H	^13^C	^1^H	^13^C	^1^H	^13^C
1		160.6		160.6		160.7
2		88.4		88.4		88.3
3		164.3		164.3		164.7
4		107.6		107.6		107.7
4a		155.2		155.2		155.2
5a		148.2		148.2		148.2
6		100.1		100.1		100.1
7		149.9		149.9		150.0
8		108.2		108.2		108.2
9		147.3		147.4		147.4
9a		112.8		112.8		112.9
9b		109.7		109.7		109.6
1′		206.3		206.3		206.2
2′	3.17 (3H, s)	42.7	3.18 (2H, t)	42.5	3.15 (2H, t)	44.7
3′	1.61 (2H, quin)	23.9	1.60 (2H, quin)	26.4	1.64 (2H, sext)	17.6
4′	1.32 (4H, m)	30.6	1 (2H, sext)	22.0	0.95 (3H, t)	13.9
5′		22.0	0.91 (3H, t)	13.9		
6′	0.89 (3H,t)	13.8				
1‐OMe						
3‐OMe						
7‐OMe	3.83 (3H, s)	60.8	3.82 (3H, s)	60.8	3.83 (3H, s)	60.8
1‐OH						
7‐OH						
9‐OH						

NMR data are shown in Fig. S2.

#### CDF‐1

^1^H NMR (dimethylsulfoxide): 0.89 (3H, t, 6′‐CH_3_), 1.32 (4H, m, 4′,5′‐CH_2_‐), 1.61 (2H, quin, 3′‐CH_2_‐), 3.17 (2H, t, 2′‐CH_2_‐), 3.83 (3H, s, 7‐OMe).

^13^C NMR (dimethylsulfoxide): 13.8 (C‐6′), 22.0 (C‐5′), 23.9 (C‐3′), 30.6 (C‐4′), 42.7 (C‐2′), 60.8 (7‐OMe), 88.4 (C‐2), 100.1 (C‐6), 107.6 (C‐4), 108.2 (C‐8), 109.7 (C‐9b), 112.8 (C‐9a), 147.3 (C‐9), 148.2 (C‐5a), 149.9 (C‐7), 155.2 (C‐4a), 160.6 (C‐1), 164.3 (C‐3), 206.3 (C‐1′).

#### CDF‐2

^1^H NMR (dimethylsulfoxide): 0.91 (3H, t, 5′‐CH_3_), 1.35 (2H,sext, 4′‐CH_2_‐), 1.60 (2H, quin, 3′‐CH_2_‐), 3.18 (2H, t, 2′‐CH_2_‐), 3.82 (3H, s, 7‐OMe).

^13^C NMR (dimethylsulfoxide): 13.9 (C‐5′), 22.0 (C‐4′), 26.4 (C‐3′), 42.5 (C‐2′), 60.8 (7‐OMe), 88.4 (C‐2), 100.1 (C‐6), 107.6 (C‐4), 108.2 (C‐8), 109.7 (C‐9b), 112.8 (C‐9a), 147.4 (C‐9), 148.2 (C‐5a), 149.9 (C‐7), 155.2 (C‐4a), 160.6 (C‐1), 164.3 (C‐3), 206.3 (C‐1′).

#### CDF‐3

^1^H NMR (dimethylsulfoxide): 0.95 (3H, t, 4′‐CH_3_), 1.64 (2H, sext, 3′‐CH_2_‐), 3.15 (2H, t, 2′‐CH_2_‐), 3.83 (3H, s, 7‐OMe).

^13^C NMR (dimethylsulfoxide): 13.9 (C‐4′), 17.6 (C‐3′),44.7 (C‐2′), 60.8 (7‐OMe), 88.3 (C‐2), 100.1 (C‐6), 107.7 (C‐4), 108.2 (C‐8), 109.6 (C‐9b), 112.9 (C‐9a), 147.4 (C‐9), 148.2 (C‐5a), 150.0 (C‐7), 155.2 (C‐4a), 160.7 (C‐1), 164.7 (C‐3), 206.2 (C‐1′).

The structures of CDF2 and 3 are shown in Fig. 4B.

### Antibacterial activity

Because CDF‐2,3 have similar structures to CDF‐1, which is known to have antibacterial activity, we tested the activity of all three CDF compounds against a panel of bacteria, including both Gram‐positive and Gram‐negative and the standard food bacteria for *Dictyostelium*. Ampicillin was used for comparison. The results of the individual antimicrobial activity tests are shown in Fig. S3‐1 and ‐2 and a summary of these results is presented in Table 4.

**Table 4:** Summary of antibacterial activities (MIC). *S. epidermidis* and *B. subtilis* are Gram‐positive bacteria. *E. coli, K. aerogenes* and *P. fluorescens* are Gram‐negative bacteria.

Tested organism	MIC (μg·mL^−1^)			
	CDF‐1	CDF‐2	CDF‐3	Ampicillin
*S. epidermidis* ATCC14990	0.098	0.19	0.39	3.75
*B. subtilis* ATCC6051	0.39	0.39	0.78	1.9
*B. subtilis*	0.19	0.78	0.78	0.94
*E. coli* B/r	6.25	12.5	12.5	3.75
*E. coli* NBRC 14249	> 100	> 100	> 100	3.75
*P. fluorescens* ATCC13525	> 100	> 100	> 100	> 120
*K. aerogenes*	> 100	> 100	> 100	120

The results of the MIC assays are clear: although the CDF compounds have limited activity against Gram‐negative bacteria, they are very potent against the two Gram‐positive species tested, *S. epidermidis* (ATCC 14990) and *B. subtilis* (ATCC 6051), being in all cases more effective than ampicillin. CDF‐1 is the most potent, and because the CDFs differ only in the length of their acyl side‐chains, this shows that side chain length is one determinant of potency.

## Discussion

In the present study, we have purified and established the chemical structures of two chlorinated compounds CDF‐2 and ‐3 made by fruiting bodies of *D. discoideum* and closely related cellular slime moulds. Our main difficulty was in obtaining sufficient material for identification. In microorganisms, secondary metabolite production is expected to be regulated by the particular environment the organism experiences: therefore, to improve yields, it pays to vary this, as was our experience. Further increases in yield came from adding zinc and propionic acid to the medium and by choosing a high‐producing species (*D. dimigraformum*). Together, these modifications gave sufficient material for NMR, which, together with MS, allowed identification.

CDF‐2 and ‐3 are chlorinated dibenzofurans, closely related to the previously identified CDF‐1 [14], being homologues with one or two fewer carbons in their acyl side chains (C5 or C4 as against C6). All three compounds are likely made by a similar biosynthetic route employing the StlB polyketide synthase and ChlA chlorinating enzyme, which are both genetically essential for their production [14]. Synthesis could start with an alkyl phenone, made by the chalcone synthase domain of the StlB PKS, which is released from the intact StlB in late development after the peak of DIF production by the enzyme. The domain functions separately for CDF production, with release expected to reduce its stringency in selecting acyl‐CoA starter units, thus allowing the production of a family of closely related compounds.

CDF‐1–3 are members of a wider family of closely related chlorinated dibenzofurans produced across Dictyostelid species. Other members include AB0022A from *D. purpureum*, which only differs from CDF‐3 by a methoxy for hydroxy substitution and Pf1 and Pf2 from *P. filamentosum* which differ in their acyl chain length and by a methoxy substitution [27, 28]. Because the polyketide synthase, StlB, and chlorinating enzyme, ChlA, which make CDF1‐3 are also conserved across Dictyostelid species, it seems likely that this family of compounds are all produced by a common pathway and have a conserved role in Dictyostelid biology.

Following on from our earlier work with CDF‐1 [14], we found that CDF‐2 and ‐3 are also potent antibacterials, with all three CDF compounds being more potent against Gram‐positive than Gram‐negative bacteria and often more potent than ampicillin. Indeed, of the Gram‐negatives tested, only *E. coli* B/r was sensitive to the CDF compounds, whereas *E. coli* NBRC14249, for instance, appears fully resistant. This strain difference could reflect the laboratory‐adaptation of *E. coli* B/r, which may have lost antibiotic‐resistance, whereas *E. coli* NBRC14249 (derived from the K‐11 strain) is used as a susceptibility reference strain for evaluating antimicrobial agents. However, comparing the *Escherichia coli* B/r and NBRC14249 genomes did not suggest any obvious differences in membrane permeability or efflux mechanisms to explain their different sensitivities.

Comparing the potency of the different chlorinated dibenzofurans against Gram‐positive bacteria allows some limited structure–function relationships to be inferred. The CDFs differ only in the length of their acyl side chain and our results suggest that their potency increases with the length of this moiety up to at least C_6_. AB0022A and CDF‐1 differ only in the substitution of a methoxy for a hydroxy group. CDF‐1 appears to be more potent than AB0022A against *Staphylococcus epidermidis* and *Bacillus subtilis*, although a caveat is that these results come from different laboratories. This suggests that the methoxy substitution in AB0022A reduces its antimicrobial activity. In summary, considered as a group, the Dictyostelid chlorinated dibenzofurans are potent antibacterials against a range of Gram‐positive bacteria, but not against Gram‐negatives. At least one (Pf1) is reported to be active against multi‐drug‐resistant *S. aureus* [30].

The stalked *Dictyostelium* fruiting body produced at the end of development consists of dead stalk cells and viable, dormant spores protected by a cellulosic coat. It is considered that the sacrifice of the amoebae making up the stalk, together with the earlier thermotactic and phototactic migration of the slug, helps disperse the spores by increasing encounters with invertebrates, which can transport them to new food sources [31]. The stalk cells of this fruiting body are the site of CDF synthesis and accumulation, which is to high micro‐molar concentrations [13]. What might be its function in Dictyostelid biology?

Because the CDFs mainly accumulate after morphogenesis is complete, they are unlikely to be developmental signals like DIF‐1. Conceivably they are inhibitors of premature spore germination, although this role is already filled by discadenine and a different polyketide [32, 33]. They might also aid spore dispersal by attracting soil invertebrates to transport them to new sites, but this does not account for their antibacterial activity.

In the light of our results and previous studies, we propose that the function of the CDF compounds is ecological: to protect the spores in the fruiting body from degradative bacteria. Supporting this, the CDF compounds have strong antibacterial activity and appear to be sufficiently abundant in fruiting bodies to be effective: they are present at high micromolar concentrations [13] but active at low micromolar concentrations. Identical or similar compounds are widely distributed among Dictyostelids, along with their biosynthetic machinery. Thus, it is conceivable that a final altruistic function of the dying stalk cells is the synthesis of protective polyketides for the dormant spores.

The present study also highlights the potential of Dictyostelid amoebae as a source of antimicrobial agents and possibly other bioactive compounds, implied by the richness of PKS genes in their genomes. The CDF compounds are most active against Gram‐positive bacteria, perhaps implying that agents against Gram‐negatives await discovery. Finally, our findings suggest that exploring both the diversity of producer species and optimising culture conditions may be important strategies for obtaining such compounds in quantities sufficient for identification.

## Conflicts of interest

The authors declare that they have no conflicts of interest.

## Author contributions

TS, TY and RRK conceived and designed the project. TY acquired the data. TY, UT, RRK and TS analysed and interpreted the data. TS and RRK wrote the paper.

## Supporting information


**Fig. S1.** CDF samples were analysed by ESI mass spectrometry with negative ion mode.
**Fig. S2.** NMR data of CDF‐1, ‐2 and ‐3.
**Fig. S3.** Results of antimicrobial activity tests 1 (Gram‐positive bacteria). 2 (Gram‐negative bacteria).

## Data Availability

Supplementary data are compiled in the Supporting information. Other data are available upon reasonable request.
