# Supplementary material for: Production of antibacterial compounds by a Steely hybrid polyketide synthase in Dictyostelium
Source: FEBS Open Bio. 2025 Oct 8;16(1):68–78. doi: 10.1002/2211-5463.70124 (PMC12767764; doi:10.1002/2211-5463.70124)
Supplement: Supplementary file 1 — Fig. S1. CDF samples were analysed by ESI mass spectrometry with negative ion mode. Fig. S2. NMR data of CDF‐1, ‐2 and ‐3. Fig. S3. Results of antimicrobial activity tests 1 (Gram‐positive bacteria). 2 (Gram‐negative bacteria). [file FEB4-16-68-s001.pdf]

Production of anti-bacterial compounds by a Steely hybrid polyketide synthase in *Dictyostelium*

Tomoaki R. Yamashita[1], Toyonobu Usuki[2], Robert R. Kay[3], Tamao Saito＊[2]

1. Graduate School of Science and Technology, Sophia University, 7-1 Kioicho, Chiyoda-ku, Tokyo 102-8554, JAPAN

2. Faculty of Science and Technology, Sophia University, 7-1 Kioicho, Chiyoda-ku, Tokyo 102-8554, JAPAN

3. MRC Laboratory of Molecular Biology, Francis Crick Avenue, Cambridge Biomedical Campus,

Cambridge CB2 0QH, UK

*Correspondence author

Tamao Saito

Faculty of Science and Technology, Sophia University, 7-1 Kioicho, Chiyoda-ku, Tokyo 102-8554, JAPAN

Tel : +81-3-3238-3366 E-mail : tasaito@sophia.ac.jp

(A)

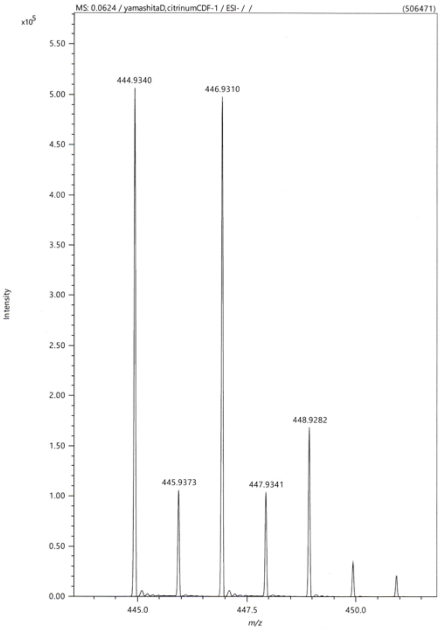
MS: 0.0624 / yamashitaD,citrinumCDF-1 / ESI- / /
(506471)
x10^5
Intensity
m/z
444.9340
446.9310
448.9282
445.9373
447.9341

(B)

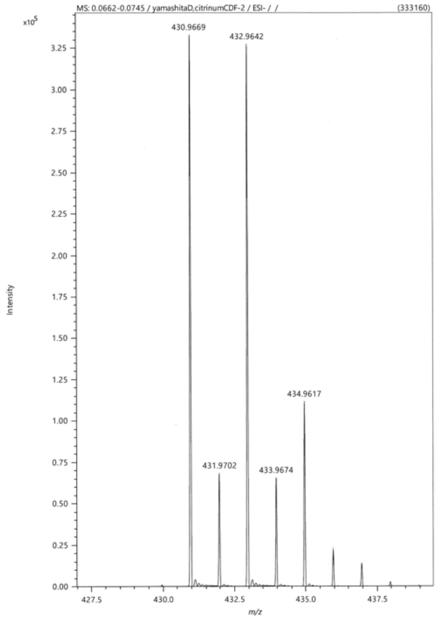
MS: 0.0662-0.0745 / yamashitaD,citrinumCDF-2 / ESI- / /
(333160)
x10^5
Intensity
m/z
430.9669
432.9642
434.9617
431.9702
433.9674

(C)

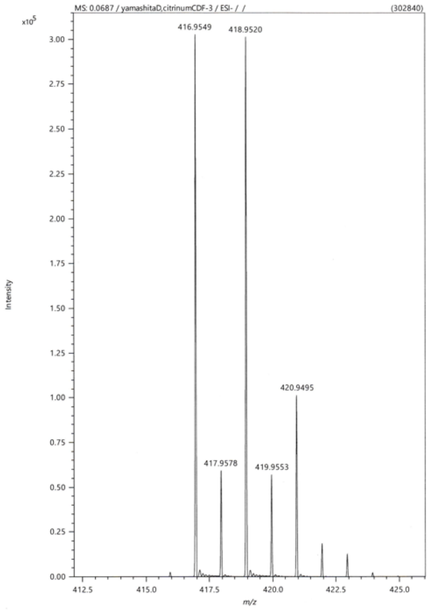
MS: 0.0687 / yamashitaD,citrinumCDF-3 / ESI- / /
(302840)
x10^5
Intensity
m/z
416.9549
418.9520
420.9495
417.9578
419.9553

(D)

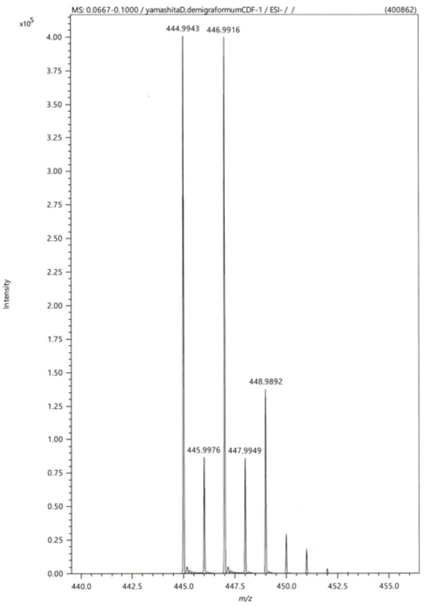
MS: 0.0667-0.1000 / yamashitaD,demigraformumCDF-1 / ESI- / /
(400862)
x10^5
Intensity
m/z
444.9943
446.9916
445.9976
447.9949
448.9892

(E)

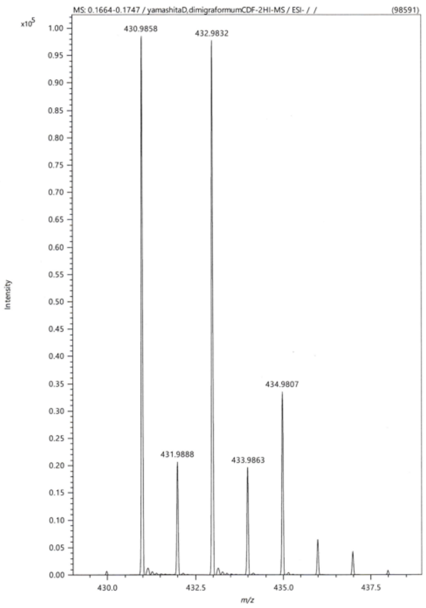
MS: 0.1664-0.1747 / yamashitaD,dimigraformumCDF-2HI-MS / ESI- / /
(98591)
x10^5
Intensity
m/z
430.9858
432.9832
431.9888
433.9863
434.9807

(F)

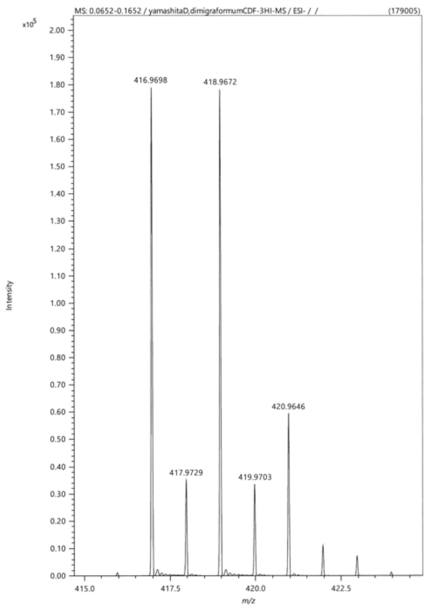
MS: 0.0652-0.1652 / yamashitaD,dimigraformumCDF-3HI-MS / ESI- / /
(179005)
x10^5
Intensity
m/z
416.9698
418.9672
417.9729
419.9703
420.9646

(G)

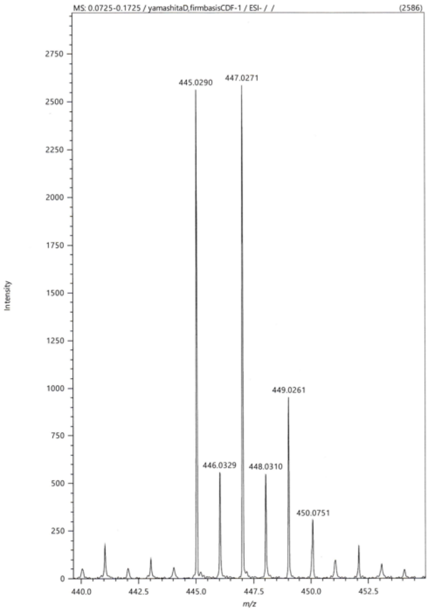
MS: 0.0725-0.1725 / yamashitaD,firmbasisCDF-1 / ESI- / /
(2586)
Intensity
m/z
445.0290
447.0271
446.0329
448.0310
449.0261
450.0751

(H)

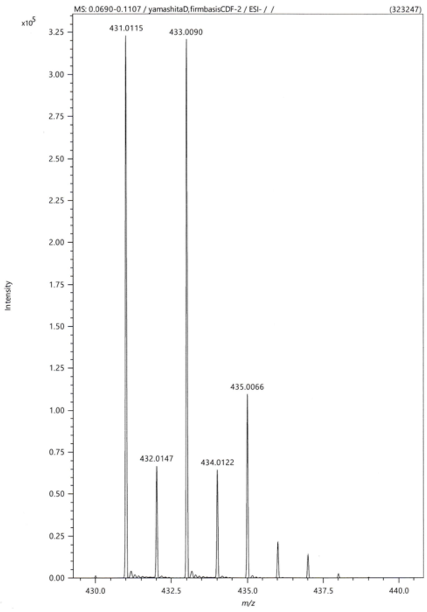
MS: 0.0690-0.1107 / yamashitaD,firmbasisCDF-2 / ESI- / /
(323247)
x10^5
Intensity
m/z
431.0115
433.0090
432.0147
434.0122
435.0066

(I)

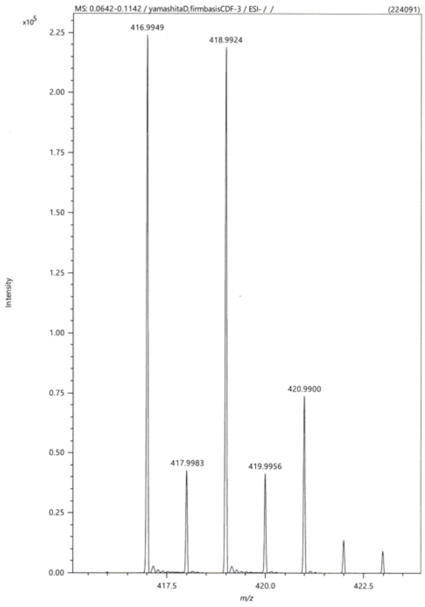
MS: 0.0642-0.1142 / yamashitaD,firmbasisCDF-3 / ESI- / /
(224091)
x10^5
Intensity
m/z
416.9949
418.9924
417.9983
419.9956
420.9900

(J)

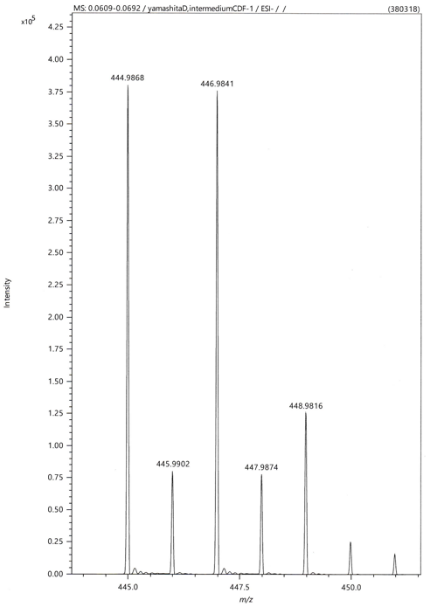

(K)

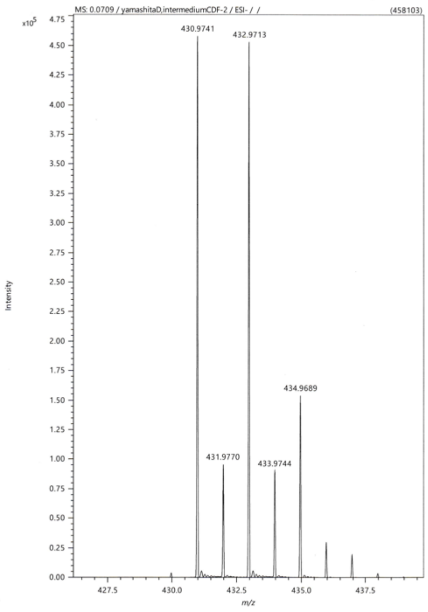

(L)

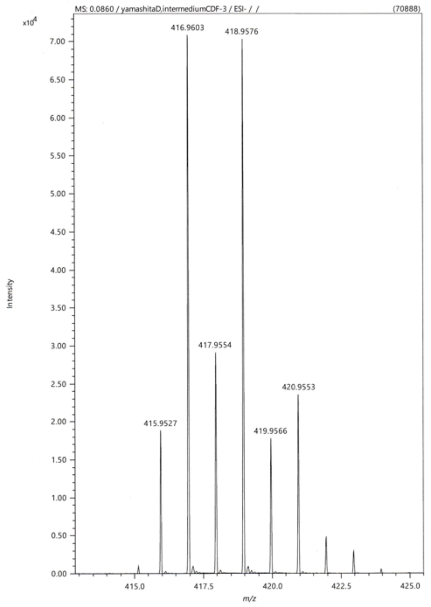

S Fig.1 CDF samples were analysed by ESI mass spectrum with negative ion mode.
CDF-1,2-3 from *D. citrinum* (A)-(C), CDF-1,2-3 from *D. dimigraformum* (D)-(F), CDF-1,2-3 from *D. firmibasis* (G)-(I), CDF-1,2-3 from *D. intermedium* (J)-(L).

(A)

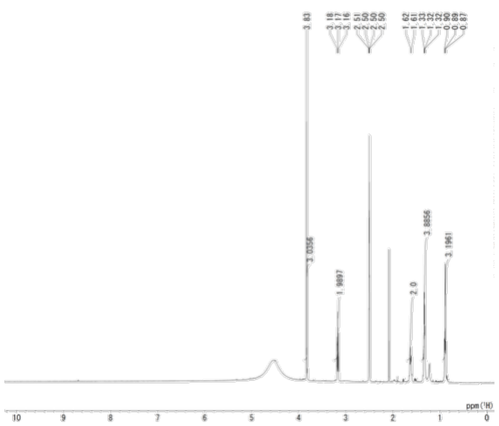

(B)

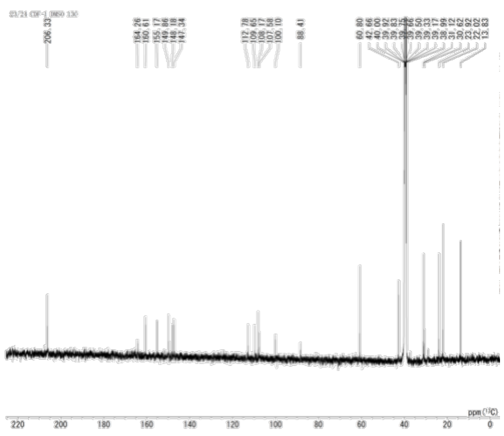

(C)

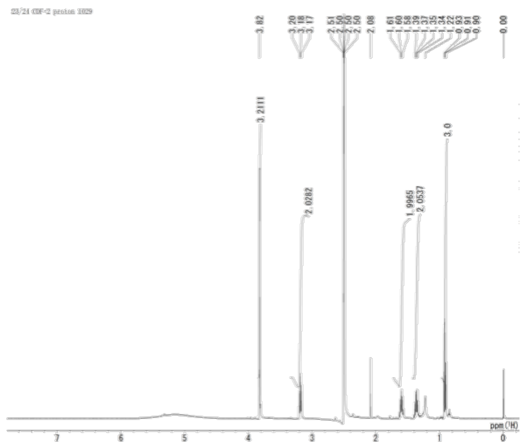

(D)

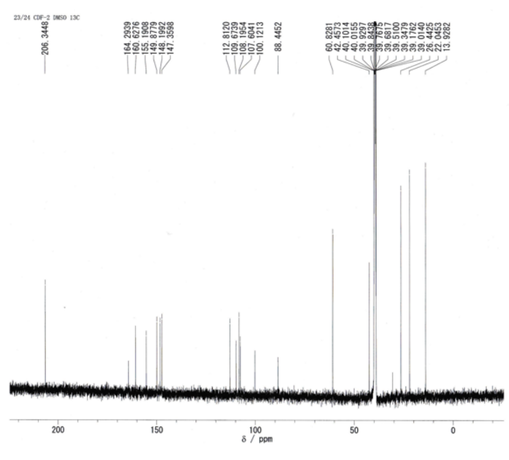

(E)

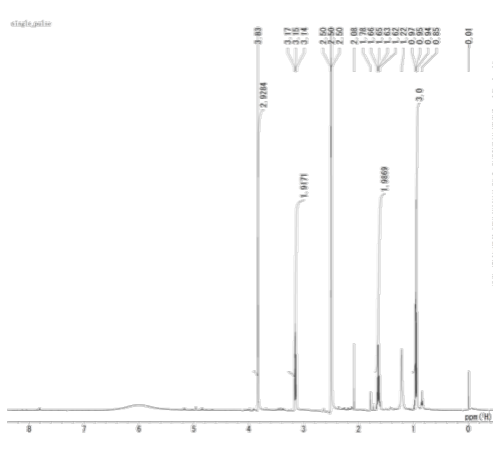

(F)

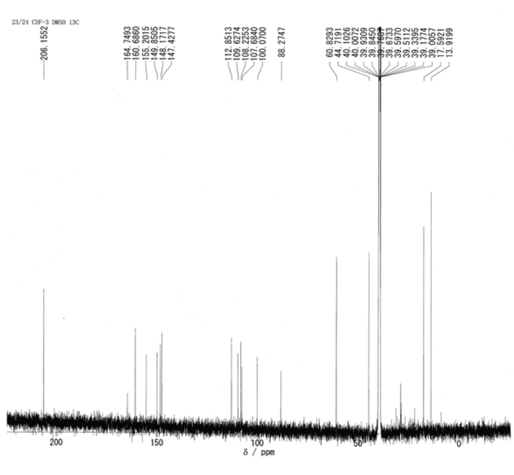

S Fig. 2 NMR data of CDF-1, -2 and -3

^{1}H-NMR of CDF-1 (A), ^{13}C-NMR of CDF-1(B), ^{1}H-NMR of CDF-2(C), ^{13}C-NMR of CDF-2(D), ^{1}H-NMR of CDF-3(E), ^{13}C-NMR of CDF-3(F).

Staphylococcus epidermidis ATCC14990

	n =1	n =2	n =3	n =4	MIC(μg/mℓ)
Amp	0.47	1.9	3.75	3.75	**3.75**
CDF-1	0.0244	0.098	0.098	0.098	**0.098**
CDF-2	0.0244	0.19	0.19	0.19	**0.19**
CDF-3	0.0488	0.39	0.19	0.39	**0.39**

Bacillus subtilis ATCC6051

	n =1	n =2	n =3	n =4	MIC(μg/mℓ)
Amp	1.9	1.9	0.94	0.94	**1.9**
CDF-1	0.19	0.19	0.39	0.19	**0.39**
CDF-2	0.39	0.39	0.39	0.39	**0.39**
CDF-3	0.39	0.39	0.78	0.78	**0.78**

Bacillus subtilis

	n = 1	n = 2	n = 3	n = 4	MIC(μg/mℓ)
Amp	0.94	0.47	0.23	0.47	**0.94**
CDF-1	0.19	0.19	0.19	0.19	**0.19**
CDF-2	0.39	0.39	0.78	0.39	**0.78**
CDF-3	0.78	0.78	0.78	0.78	**0.78**

S Fig. 3-1 Results of antimicrobial activity tests （Gram positive bacteria）

Amp refers to ampicillin. The highest concentration in each trial was determined as the MIC.

Escherichia coli B/r

	n =1	n = 2	n =3	MIC(μg/mℓ)
Amp	1.9	3.75	3.75	**3.75**
CDF-1	6.25	6.25	6.25	**6.25**
CDF-2	12.5	12.5	12.5	**12.5**
CDF-3	12.5	12.5	12.5	**12.5**

Escherichia coli NBRC 14249

	n =1	n = 2	n =3	MIC(μg/mℓ)
Amp	3.75	3.75	1.9	**3.75**
CDF-1	>100	>100	>100	**>100**
CDF-2	>100	>100	>100	**>100**
CDF-3	>100	>100	>100	**>100**

Pseudomonas fluorescens ATCC13525

	n =1	n = 2	n =3	MIC(μg/mℓ)
Amp	＞120	＞120	＞120	**＞120**
CDF-1	>100	>100	>100	**>100**
CDF-2	>100	>100	>100	**>100**
CDF-3	>100	>100	>100	**>100**

Klebsiella aerogenes

	n =1	n =2	n = 3	MIC(μg/mℓ)
Amp	120	120	120	**120**
CDF-1	>100	>100	>100	**>100**
CDF-2	>100	>100	>100	**>100**
CDF-3	>100	>100	>100	**>100**

S Fig. 3-2 Results of antimicrobial activity tests （Gram negative bacteria）

Amp refers to ampicillin. The highest concentration in each trial was determined as the MIC.
